# 

**Published:** 1889-08-17

**Authors:** 


SATURDAY, AUGUST 17th, 1889.

**The Maybrick Case: Lawyers v. Doctors** - 303
**Words of Consolation**—The Love of Man to Man - 304
**On Some Points Connected with Cholera** - 305
**The London Hospital and the National Pension Fund for Nurses** - 306
**The Doctor's Pulpit.**—In Nature's School - 307
**Children's Country Holidays Fund.**—The Poorest Children - 308
**Within the Wards.**—Foreign Bodies in Bronchial Tubes - 308
**The Story of the Insane from Year to Year.**—Asylum Reports - 309
**Notes and News** - 310
**Everybody's Page** - 312
**Annotations.**—7,000 Made Happy—Palpitation—Awake at Last - 313
**The Plans and Purposes of the Johns Hopkins Hospital.**—I. By JOHN S. BILLINGS, M.D., Surgeon, U.S. Army - 314
**Her Heart's Mistake.** By E. D. CUMMING. Chap. xviii. 315
**The Nursing in Fever Hospitals** - 317
**The Student's Column.**—University of London - 317
**Editor's Letter-Box.**—What Should the Deaf Do? - 318
**Amusements and Relaxation** - 318
**Scraps and Gleanings** - 318

**EXTRA SUPPLEMENT—THE NURSING MIRROR.**

**En Passant.**—Doll Competition—Vancouver—Sisters' Uniform—Salvation Army Nurses—Ergot—Visitors to Cambridge—Work in India—Nursing at Ashton - lxxv
**Physiology for Nurses.** No. 6.—On the Skeleton - lxxvi
**The Nurses' Home Sydney** - lxxvii
**Everybody's Opinion.**—The Pension Fund—The Sunderland Sisters—Asylum Attendants - lxxvii
**Winter Amusements and Remunerative Work.**—An Exhibition of Doll Nurses - lxxviii
**Appointments—Vacancies—Notes and Queries** lxxviii

